# Syntheses of two deacetyl-thymosin α_1_ analogues and their effects on low E-rosette-forming lymphocytes of uraemic patients

**DOI:** 10.1155/S0962935196000439

**Published:** 1996-09

**Authors:** T. Abiko, H. Sekino

**Affiliations:** 1 Kidney Research Laboratory Kojinkai, 1-6 Tsutsujigaoka 2-chome Miyagino-ku Sendal 980 Japan

## Abstract

Two deacetyl-thymosin α^1^ analogues containing Phe (4Br) or D-Phe (4Br) residue—[D-Phe(4Br)^21^]deacetyl-thymosin α_1_ and [Phe(4Br)^21^]deacetyl-thymosin α_1_, respectively—were synthesized by the manual solid-phase method and their immunological effects on the low E-rosette-forming lymphocytes of uraemic patients were examined. One of the synthetic analogues, [Phe(4Br)^21^deacetyl-thymosin α_1_, demonstrated a restorative effect on the low E-rosette-forming lymphocytes of uraemic patients, which was stronger than that of deacetyl-thymosin α_1_, but the other analogue, [D-Phe(4Br)^21^]deacetyl-thymosin α_1_, showed no restorative effect under the same conditions.


Mediators of Inflammation 5, 295–298 (1996)

### Syntheses of two deacetyl-thymosin $\alpha_1$ analogues and their effects on low E-rosette-forming lymphocytes of uraemic patients

T. Abiko[CA] and H. Sekino

Kidney Research Laboratory, Kojinkai, 1-6 Tsutsujigaoka 2-chome, Miyagino-ku, Sendai 980, Japan

[CA]Corresponding Author

**Two deacetyl-thymosin $\alpha^1$ analogues containing Phe (4Br) or D-Phe (4Br) residue—[D-Phe(4Br)$^{21}$]-deacetyl-thymosin $\alpha_1$ and [Phe(4Br)$^{21}$]deacetyl-thymosin $\alpha_1$, respectively—were synthesized by the manual solid-phase method and their immunological effects on the low E-rosette-forming lymphocytes of uraemic patients were examined. One of the synthetic analogues, [Phe(4Br)$^{21}$deacetyl-thymosin $\alpha_1$, demonstrated a restorative effect on the low E-rosette-forming lymphocytes of uraemic patients, which was stronger than that of deacetyl-thymosin $\alpha_1$, but the other analogue, [D-Phe(4Br)$^{21}$]deacetyl-thymosin $\alpha_1$, showed no restorative effect under the same conditions.**

**Key words:** Deacetyl-thymosin $\alpha_1$ analogue synthesis, Impaired immunological function, Low E-rosette-forming lymphocyte, Restorative effect, Uraemic patient

### Abbreviations

Z, benzyloxycarbonyl; OBzl, benzyl ester; Boc, tert-butoxycarbonyl; DCC, N,N′-dicyclohexylcarbodiimide; HOBT, N-hydroxybenzotriazole; DMF, dimethylformamide; E-rosette, a rosette with sheep erythrocytes; TFA, trifluoroacetic acid; Phe(4Br), *p*-bromophenylalanine; $Et_3N$, triethylamine; AcOH, acetic acid; PBS, phosphate-buffered saline; $GVB^{2+}$, gelatin veronal buffer; HPLC, high-performance liquid chromatography; TLC, thin-layer chromatography; Ac, acetyl; EtOH, ethanol; FAB-MS, fast atom bombardment mass spectrometry; Bzl, benzyl.

### Introduction

Thymosin $\alpha_1$, a peptide containing 28 amino acid residues, was isolated by Goldstein *et al.*[1] from thymosin fraction 5, a mixture of peptides from calf thymus. Thymosin $\alpha_1$ was found to be active in some of the *in vitro* tests used for thymosin fraction 5,[2] and it was considered to be one of the factors that modulated steps in the maturation of T-lymphocytes.[1,2]

In our earlier papers,[3,4] we have demonstrated that not only our synthetic deacetyl-thymosin $\alpha_1$ but also its two synthetic analogues, [Phe$^{21}$]deacetyl-thymosin $\alpha_1$ and [Phe(4F)$^{21}$]deacetyl-thymosin $\alpha_1$ exhibit restorative activity on low E-rosette-forming lymphocytes of uraemic patients. The synthetic [Phe$^{21}$]deacetyl-thymosin $\alpha_1$ was approximately equal in potency to our synthetic deacetyl-thymosin $\alpha_1$ in uraemic patients.[4] We also found that the acetyl group at the N-terminal Ser residue of thymosin $\alpha_1$ is not required for restorative activity of low E-rosette-forming lymphocytes.[4] Interestingly, fluorination of the para-position of Phe (position 21) resulted in a marked restorative effect on the low E-rosette-forming lymphocytes of uraemic patients compared with that of [Phe$^{21}$]deacetyl-thymosin $\alpha_1$.[4]

These results prompted us to synthesize two deacetyl-thymosin $\alpha_1$ analogues containing brominated aromatic rings which influence electron-intensity on aromatic rings such as fluorinated ones.

This paper deals with the solid-phase syntheses of [D-Phe(4Br)$^{21}$]deacetyl-thymosin $\alpha_1$ and [Phe(4Br)$^{21}$]deacetyl-thymosin $\alpha_1$ and an examination of the immunological effect of two analogues, [Phe(4F)$^{21}$]deacetyl-thymosin $\alpha_1$ and deacetyl-thymosin $\alpha_1$, on the low E-rosette-forming lymphocytes of uraemic patients.[3,4]

### Materials and Methods

*Resin, amino acid derivatives and reagents:* The chloromethylated polystyrene resin crosslinked with 2% divinylbenzene (Cl content 2 mmol/g) and Boc-amino acids, Boc-Ser(Bzl)-OH, Boc-Asp(OBzl)-OH, Boc-Ala-OH, Boc-Val-OH, Boc-Thr(Bzl)-OH, Boc-Glu(OBzl)-OH, Boc-Ile-OH, Boc-Lys(Z)-OH, Boc-Leu-OH, Boc-Asn-OH, Boc-D-Phe(4Br)-OH and Boc-Phe(4Br)-OH, were

```
                        5                   10                  15
Ac-Ser-Asp-Ala-Ala-Val-Asp-Thr-Ser-Ser-Glu-Ile-Thr-Thr-Lys-Asp-

                20                  25
Leu-Lys-Glu-Lys-Lys-Glu-Val-Val-Glu-Glu-Ala-Glu-Asn-OH
```

FIG. 1. Amino acid sequence of thymosin $\alpha_1$.

© 1996 Rapid Science Publishers purchased from Protein Research Inc. (Mino, Osaka, Japan), Kokusan Chemical Works Ltd (Kyoto, Japan) and Bachem Feinchemikalain AG. Solvents were freshly distilled. The purified synthetic [D-Phe(4Br)[21]]deacetyl-thymosin $\alpha_1$ and [Phe-(4Br)[21]]deacetyl-thymosin $\alpha_1$ were chromatographed on cellulose plates (Merck). $Rf^1$ values refer to BuOH-AcOH-$H_2O$ (4:1:5, upper layer) and $Rf^2$ values refer to BuOH-pyridine-AcOH-$H_2O$ (30:20:6:24). Amino acid analysis was performed by the Hitachi Model 835-50 amino acid analyser. HPLC was conducted with a Shimadzu LC-9A apparatus coupled to an analytical YMC-AM-312 column (6.0 × 150 cm). FAB-MS spectrum was obtained on a Auto Spec Q with an OPUS data processor.

*Patient selection:* Four uraemic patients who were suffering from recurrent infectious diseases were selected. Venous blood was obtained from these uraemic patients for the E-rosette formation test. Venous blood samples from two healthy donors were used as a control.

*Solid-phase peptide synthesis:* Solid-phase peptide syntheses of [D-Phe(4Br)[21]]deacetyl-thymosin $\alpha_1$ and [Phe(4Br)[21]]deacetyl-thymosin $\alpha_1$ were carried out manually in a glass vessel by a stepwise strategy starting with Boc-Asn-resin (0.18 mmol/g, 1 g).

The general procedure for each synthetic cycle was: (1) three washings with $CH_2Cl_2$; (2) prewashing with 40% TFA in $CH_2Cl_2$; (3) deprotection for 30 min with TFA in $CH_2Cl_2$; (4) three washings with $CH_2Cl_2$ (5) prewashing with $Et_3N$ in $CH_2Cl_2$; (6) neutralization for 10 min with 10% $Et_3N$ in $CH_2Cl_2$; (7) three washings with $CH_2Cl_2$; (8) addition of 4 eq Boc-amino acid, HOBT and DCC respectively; (9) reaction for 120 min in DMF or N-methyl-2-pyrrolidone + DMF (1:1); (10) three washings each with $CH_2Cl_2$, 50% EtOH in $CH_2Cl_2$, DMF and then $CH_2Cl_2$. Double couplings were done when necessary as judged by the ninhydrin test. A triple coupling was only necessary for Ile; (11) 0.4 M acetylimidazole in DMF (1 ×, 30 min); (12) two washings with DMF and two washings with $CH_2Cl_2$.

The protected peptide resin was treated with anhydrous hydrogen fluoride containing 10% anisole at 0°C for 60 min. After evaporation of excess hydrogen fluoride under vacuum, the crude peptide was extracted with 10% AcOH and the extract was washed with ether and then evaporated to dryness under vacuum.

*Purification of deprotected [D-Phe(4Br)[21]]deacetyl-thymosin $\alpha_1$:* The deprotected crude octaeicosapeptide was dissolved in $H_2O$ (5 ml). The solution was treated with Amberlite CG-4B (acetate form, approximately 2 g) for 30 min and filtered by suction. The filtrate was adjusted to pH 8.0 with 1 N $NH_4OH$ and stirred in an ice-bath for 30 min to reverse a possible N → O shift at the Ser and Thr residues. The pH of the solution was adjusted to pH 6.0 with a few drops of 1 N AcOH, and the solution was lyophilized. The residue was dissolved in 2% AcOH (2 ml), applied to a column of Sephadex G-25 (2.8 x 97 cm), and eluted with the same solvent. Fractions of 4 ml each were collected per 15 min, and the absorption at 260 nm was determined (see Fig. 2). Fractions corresponding to the front main peak (tube nos 56–64) were combined and the solvent was removed by evaporation. Analysis by TLC revealed the presence of three ninhydrin-positive spots with $Rf^1$ 0.11 (main), 0.28 (minor) and 0.36 (minor). The crude product was dissolved in a small amount of water and subjected to preparative TLC (cellulose plate, 20 × 40 cm) using the upper layer of BuOH-AcOH-$H_2O$ (4:1:5) as a developing solvent. The zone corresponding to $Rf^1$ 0.11 was separated and extracted with 2% AcOH. The extracts were concentrated, applied to a column of Sephadex G-25 (2.8 x 97 cm), and aluted with 2% AcOH as already described: yield 5.6% based on the starting resin, $[\alpha]_D^{21}$ – 80.9° ($c$ = 0.4, 2% AcOH), $Rf^1$ 0.11, $Rf^2$ 0.15, single ninhydrin- and chlorine-tolidine positive spot. The synthetic peptide exhibited a single spot on a paper electrophoresis; Toyo Roshi No. 51 (2 × 40 cm), pyridinium-acetate buffer at pH 7.3, mobility 2.5 cm from the origin toward the anode after running at 2 mA, 650 V for 90 min. Amino acid ratios in a 6 N HCl hydrolysate: Ile 1.00, Ala 3.03, Leu 1.01, Val 1.94, Ser 2.85, Thr 2.88, D-Phe(4Br) 0.93, Asp 3.90, Glu 5.87, Lys 4.02 (recovery of Ile 86%). The synthetic peptide

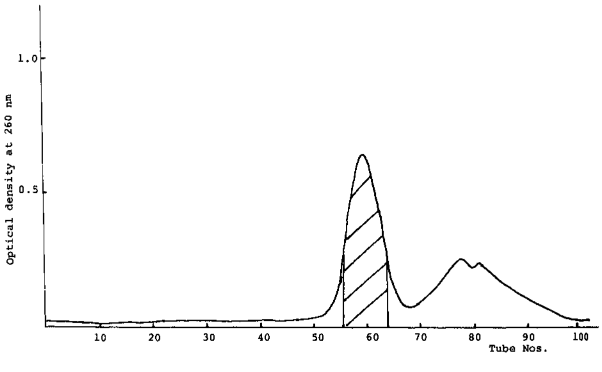

FIG. 2. Purification of synthetic [D-Phe(4Br)[21]]deacetyl-thymosin $\alpha_1$ by gel-filtration chromatography on a Sephadex G-25 column.

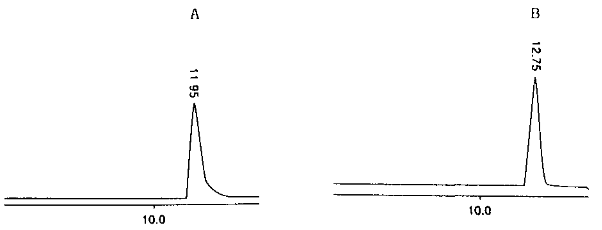

FIG. 3. HPLC profiles of (A) [D-Phe(4Br)$^{21}$]deacetyl-thymosin $\alpha_1$ and (B) [Phe(4Br)$^{21}$]deacetyl-thymosin $\alpha_1$.

exhibited a single peak on HPLC using an analytical YMC AM-312 column (6.0 × 150 mm) at a retention time of 11.95 min (A), when eluted with a gradient of acetonitrile 25 to 45% in 0.1% TFA at a flow rate of 1 ml per min (Fig. 3). FAB-MS m/z: calculated $(M + H)^+$ 3163, observed $(M + H)^+$ 3162.

*Purification of deprotected [Phe(4Br)$^{21}$]deacetyl-thymosin* $\alpha_1$: The purification procedure of the deprotected crude [Phe(4Br)$^{21}$]deacetyl-thymosin $\alpha_1$ was essentially the same as that described for the purification procedure of [D-Phe(4Br)$^{21}$]deacetyl-thymosin $\alpha_1$: yield 6.9% based on the starting resin, $[\alpha]_D^{21}$ − 91.6° ($c$ = 0.4, AcOH), $Rf^1$ 0.11, $Rf^2$ 0.14, single ninhydrin- and chlorine-tolidine positive spot. The synthetic peptide exhibited a single spot on a paper electrophoresis: Toyo Roshi No. 51 (2 × 40 cm), pyridinium-acetate buffer at pH 7.3, mobility 2.5 cm from the origin toward the anode after running at 2 mA, 650 V for 90 min. Amino acid ratios in a 6 N HCl hydrolysate: Ile 1.00, Ala 3.03, Leu 0.97, Val 1.94, Ser 2.89, Thr 2.87, Phe(4Br) 0.96, Asp 3.92, Glu 5.91, Lys 4.02 (recovery of Ile 84%). The synthetic peptide exhibited a single peak on HPLC using an analytical YML AM-312 column (6.0 × 150 mm) at a retention time of 12.75 min, when eluted with a gradient of acetonitrile 25 to 45% in 0.1% TFA at a flow rate of 1 ml per min (Fig. 3). FAB-MS m/z: calculated $(M + H)^+$ 3163, observed $(M + H)^+$ 3164.

*E-rosette formation test:* A 5-ml aliquot of venous blood was drawn into a syringe containing 300 U heparin and incubated with the synthetic peptide for 70 min at 37°C. Lymphocytes were then isolated in a Hypaque-Ficoll gradient. Isolated lymphocyte were adjusted to 5 × $10^5$ cell/ml with PBS. Contamination by monocytes and polymorphonuclear cells amounted to less than 7%. Sheep erythrocytes (Kyokuto Pharmaceutical Co.) were washed with PBS, and a suspension (1 × $10^7$ cll/ml) was prepared. The lymphocytes were washed with $GVB^{2+}$, centrifuged for 10 min at 1500 rpm, and then suspended in $GVB^{2+}$ (1 ml). The suspension was mixed with the suspension of sheep erythrocytes (0.5 ml) and incubated for 12 h at 4°C. The mixture was then centrifuged for 5 min at 900 rpm. Triplicate wet-cell preparations were checked by phase-contrast microscopy. For each preparation, 200 lymphocytes were counted, and the proportion binding more than three erythrocytes was determined.

### Results and Discussion

*In vitro* incubation of human peripheral blood lymphocytes from uraemic patients suffering from impaired immunological function with thymosin $\alpha_1$ increases the percentage of E-rosette formation.[3,4] In our previous paper,[4] we reported that incubation of peripheral venous blood lymphocytes from uraemic patients in the presence of various amounts of the two synthetic analogues, [Phe(4F)$^{21}$]deacetyl-thymosin $\alpha_1$ and [Phe$^{21}$]deacetyl-thymosin $\alpha_1$, from 0.1 to 10.0 μg/ml resulted in the recovery of E-rosette formation. Our synthetic [Phe$^{21}$]deacetyl-thymosin $\alpha_1$ was approximately equal in potency to that of our synthetic deacetyl-thymosin $\alpha_1$. Interestingly, our synthetic [Phe(4F)$^{21}$]deacetyl-thymosin $\alpha_1$ showed stronger restorative effects than that of our synthetic [Phe$^{21}$]deacetyl-thymosin $\alpha_1$.

This result encouraged us to synthetize two deacetyl-thymosin $\alpha_1$ analogues containing brominated Phe or D-Phe residue (position 21) which also have an electron-withdrawing effect on aromatic rings such as fluorinated aromatic rings.

[D-Phe(4Br)$^{21}$]deacetyl-thymosin $\alpha_1$ and [Phe$^{21}$]deacetyl-thymosin $\alpha_1$ were synthetized by the usual solid-phase method starting with Boc-Asn-Merrifield resin. The peptides were constructed on a Merrifield resin by using a single coupling reaction for each Boc-amino acid and was cleaved from the resin and protecting groups were also simultaneously deprotected by treatment with an HF-anisole mixture. Purification of the peptides was achieved by gel-filtration on a Sephadex G-25 column and preparative TLC, and we finally obtained highly purified peptides by analytical HPLC. Addition to analytical HPLC, integrity of the purified peptides was checked by TLC, amino acid analysis after 6 N HCl hydrolysis and FAB-MS spectrometry. The relative potencies of the synthetic deacetyl-thymosin $\alpha_1$ and its three analogues, [Phe(rF)$^{21}$]deacetyl-thymosin $\alpha_1$, [D-Phe(4Br)$^{21}$]deacetyl-thymosin $\alpha_1$ and [Phe(4Br)$^{21}$]deacetyl-thymosin $\alpha_1$ on restorative effect on the low E-rosette-forming lymphocytes of uraemic patients are showed in Tables 1 and 2. In contrast to non-uraemics, the percentage of E-rosette-forming lymphocytes in uraemic patients suffering from recurrent infec-

**Table 1.** Effects of synthetic deacetyl-thymosin $\alpha_1$ and its four analogues on the low E-rosette-forming capacity of lymphocytes of uraemic patients

| | Peptide | Dose (μg/ml) | No of determinations | E-rosette-forming lymphocytes[d] (%) |
|---|---|---|---|---|
| I | —[a] | — | 3 | 76 ± 5 |
| II | —[b] | — | 3 | 35 ± 4[e] |
| III | deacetyl-thymosin $\alpha_1$[b,c] | 0.1 | 3 | 37 ± 4 |
| IV | deacetyl-thymosin $\alpha_1$[b,c] | 1.0 | 3 | 54 ± 5[f] |
| V | deacetyl-thymosin $\alpha_1$[b,c] | 10.0 | 3 | 66 ± 5[f] |
| VI | [Phe$^{21}$]deacetyl-thymosin $\alpha_1$[b,c] | 10.0 | 3 | 65 ± 5[f] |
| VII | [Phe(4F)$^{21}$]deacetyl-thymosin $\alpha_1$[b,c] | 0.1 | 3 | 57 ± 5[f] |
| VIII | [Phe(4F)$^{21}$]deacetyl-thymosin $\alpha_1$[b,c] | 1.0 | 3 | 67 ± 5[f] |
| IX | [Phe($Br)$^{21}$]deacetyl-thymosin $\alpha_1$[b,c] | 0.2 | 3 | 55 ± 4[f] |
| X | [Phe(4Br)$^{21}$]deacetyl-thymosin $\alpha_1$[b,c] | 2.0 | 3 | 65 ± 4[f] |
| XI | [D-Phe(4Br)$^{21}$]deacetyl-thymosin $\alpha_1$[b,c] | 10.0 | 3 | 37 ± 5 |
| XII | [D-Phe(4Br)$^{21}$]deacetyl-thymosin $\alpha_1$[b,c] | 20.0 | 3 | 36 ± 4 |

[a]Normal venous blood. [b]Patient's venous blood.
[c]Incubation was carried out at 37°C for 70 min.
[d]Each value represents the mean ± SD of triplicate measuremetns.
[e]The significance of differences of mean values was analysed by means of Student's t-test. $p < 0.02$ compared with I.
[f]The significance of differences of mean values was analysed by means of Student's t test. $p < 0.01$ compared with II.

**Table 2.** Relative potencies of the synthetic deacetyl-thymosin $\alpha_1$ and its analogues on restorative effect on the low E-rosette-forming lymphocytes of uraemic patients

| Peptide | Relative potency[a] (molar basis) |
|---|---|
| deacetyl-thymosin $\alpha_1$ | 1.00 |
| [Phe$^{21}$]deacetyl-thymosin $\alpha_1$ | 0.98 |
| [Phe(4F)$^{21}$]deacetyl-thymosin $\alpha_1$ | 10.11 |
| [Phe(4Br)$^{21}$]deacetyl-thymosin $\alpha_1$ | 4.86 |
| [D-Phe(4Br)$^{21}$]deacetyl-thymosin $\alpha_1$ | –[b] |

[a]Relative potencies to deacetyl-thymosin $\alpha_1$ on molar basis.
[b]No effect at a dose of 20 μg/ml.

tious diseases is significantly reduced. Incubation of lymphocytes from uraemic patients in the presence of synthetic peptides (0.1–10 μg/ml) or in the absence of synthetic peptides was carried out to investigate the recovery of E-rosette-forming lymphocytes.

Our synthetic [Phe(4Br)$^{21}$]deacetyl-thymosin $\alpha_1$ showed stronger restorative activity than those of our synthetic deacetyl-thymosin $\alpha_1$ and [Phe$^{21}$]deacetyl-thymosin $\alpha_1$, but weaker than that of [Phe(4F)$^{21}$]deacetyl-thymosin $\alpha_1$. However, our synthetic [D-Phe(4Br)$^{21}$]deacetyl-thymosin $\alpha_1$ had no effect on the low E-rosette-forming lymphocytes of these patients at a dose of 20 μg/ml (Table 1).

In conclusion, the present study demonstrates that electron-withdrawing halogen atoms on the para position of aromatic rings result in analogues that possess stronger activity than that of [Phe$^{21}$]deacetyl-thymosin $\alpha_1$. The analogue having Phe(4Br) was highly active and its relative potency was just an intermediate between those of [Phe(4F)$^{21}$]deacetyl-thymosin $\alpha_1$ and deacetyl-thymosin $\alpha_1$ (Table 2). This further indicates that the electron-withdrawing effect of bromine is weaker than that of fluorine. Therefore, the incorporation of bromide atom into the para position induces a stronger interaction between Phe$^{21}$ and receptor, but it seems to be weaker than the interaction between the para-fluorinated phenylalanine$^{21}$ and receptor. On the other hand, the replacement of Phe(4Br) with D-Phe (4Br) resulted in a sharp reduction in the restorative effect on the low E-rosette-forming lymphocytes of uraemic patients. This result would seem to suggest that the L-type steric configuration at the 21st residue of thymosin $\alpha_1$ is necessary to restore immunological activity on the impaired immunological function.

### References

1. Goldstein AL, Low TLK, McAdoo M, McClure J, Thurman GB, Rossio J, Lai CY, Chang D, Wang SS, Harvey C, Ramel AH, Meinhofer J. Thymosin $\alpha_1$: isolation and sequence analysis of an immunologically active thymic peptide. *Proc Natl Acad Sci USA* 1977; **74:** 725–729.
2. Low TLK, Goldstein AL. The chemistry and biochemistry of thymosin. II. Amino acid sequence analysis of thymosin $\alpha_1$ and polypeptide $\beta_1$. *J Biol Chem* 1979; **254:** 987–995.
3. Abiko T, Sekino H. Deacetyl-thymosin $\alpha_1$: synthesis and immunological effect on lipoid nephrosis lymphocytes. *Chem Pharm Bull* 1982; **30:** 1776–1783.
4. Abiko T, Sekino H. Solid-phase syntheses of two deacetyl-thymosin $\alpha_1$ analogs with substitution at position 21 and their effects on low E-rosette-forming lymphocytes of uraemic patients. *Biotechnology Therapeutics* 1992; **3:** 159–168.

**Received 11 June 1996; accepted 26 June 1996**
